# Lay Theories of Gender Identity Disorder

**DOI:** 10.1080/00918369.2013.819208

**Published:** 2013-09-23

**Authors:** Adrian Furnham, Radhika Sen

**Affiliations:** Department of Clinical, Educational, and Health Psychology, University College London, London, UK

**Keywords:** gender identity disorder, lay theories

## Abstract

This study examined lay theories regarding gender identity disorder (GID). Pilot interviews were completed with participants (n = 10) regarding their views on possible causes and treatments of GID. Participants (mainly young British people and students; n = 124) then completed a questionnaire that was based on the interviews and a review of the salient literature on lay theories. As hypothesized, participants believed most in biomedical causes and treatments of GID. Factor analysis (with varimax rotation) identified 4 factors in relation to *causes of GID: upbringing and personal factors, pregnancy and brain abnormalities, environmental factors, and biomedical causes*. Five factors that were identified in relation to the *cure/treatment* of GID were *psychological assistance and personal factors, extreme medical and behavioral changes, alternative therapies, external factors*, and *medical treatments*. The results indicated that participants neither agreed nor strongly disagreed about causes and cures regarding GID, but that these beliefs were logically related. Limitations, particularly of sampling, were considered.

Lay theories are “the informal, ‘common sense’ explanations that people provide for particular social behaviours, and they often differ significantly from ‘scientific’ explanations and theories” (Furnham, 1988, p. 6). Understanding of the cause and nature of any mental disorder impacts on whether individuals hold stigmatizing beliefs regarding a particular disorder, as well as if, when, and why they seek professional help and advice when they are mentally unwell and whether they will adhere to the treatment provided (Haslam & Ernst, 2002). Because people with a wide range of psychological problems and conditions (i.e., schizophrenia) experience prejudice and discrimination, lay theory studies have attempted to understand the nature of lay beliefs of various conditions. Studies suggest “gender outlaws” attract discrimination (Willoughby et al., 2010), which is partly the motivation for this study. Up to now it seems that no research has been done on GID, and this study, conducted in the United Kingdom, attempts to rectify this.

Lay theory studies have shown that for many different mental illnesses, the public prefer psychosocial over biological explanations for the problem and favor self-help over more standard psychiatric treatment. Some take a “psychological essentialism” position, others take a “biological reductionist” position, and some take a “moral free choice” position on many illnesses. Furnham and Telford (2012) reviewed 24 lay theory studies of psychological problems, such as alcoholism, anorexia nervosa, and autism, to schizophrenia and suicide. Nearly all studies on the related area of mental health literacy show that the public have great difficulty in identifying many disorders (Jorm, 2000; Jorm, Angermeyer, & Katschnig, 2000; Jorm, Christensen, & Griffiths, 2005; Jorm, Nakane, et al., 2005).

Examining lay beliefs regarding a particular disorder can display how the publics’ beliefs deviate from professional practices and suggest measures for public education (Giosan, Glovsky, & Haslam, 2001). Indeed, the study of beliefs about psychiatric phenomena from a lay perspective has a long and rich history, and is now often called psychiatric or “mental health literacy.” Jorm (2000), who has done much important work in this area, has shown it to be important because it relates to sensible help-seeking behavior and the stigmatization of the mentally ill. This study is also one of a series of studies in the mental health literacy tradition looking at lay theories of specific mental illness of which the latest was psychopathy (Furnham, Daoud, & Swami, 2009) and bipolar disorder (Furnham & Anthony, 2010).

It seems no lay theory research had been carried out in relation to gender identity disorder (GID). There is an interesting and extensive literature on cultural and individual differences of correlates of attitudes toward gender (Jayaratne & Anderson, 2001; Knox, Zusman, & McNeeley, 2004; Mahalingham, 2003, 2007a, 2007b; Quinn & Luttrell, 2004; Ridgeway & Correll, 2004; Tucker & Keil, 2001). These studies have identified consistent race and cultural differences, but also class, caste, and gender differences, in beliefs about the origins of gender (as opposed to sex). This study is, however, uniquely concerned with beliefs about GID. It should be made clear that there are a number of “psychological conditions,” like transgenderism, transsexualism, gender dysphoria, gender incongruence, and gender nonconforming behavior, that overlap in terms of certain behavioral manifestations. Further, people may well react very differently to GID in childhood, adolescence, and adulthood. These distinctions are debated by academics and practitioners and are often confusing to the lay public. A simple definition of GID was given to participants in this study to help with a clear definition (see Study 2).

## PUBLIC INTEREST IN GID

In recent decades, the general population has become more aware of GID mainly through the media, but also in their daily lives, as affected individuals have more readily sought professional help, openly cross-dressed, and disclosed the details of their disorder (Zucker, 2005). The 1999 film, *Boys Don't Cry* (Vachon, Kolodner, & Peirce), was one such example of the infiltration of GID into the mass media (Di Ceglie, 2000). Many groups interested in psychological conditions form associations and have Web sites aimed to educate the public interested in the specific condition.

## PSYCHOLOGICAL THEORIES OF GID

The diagnosis of GID, according to the *Diagnostic and Statistical Manual of Mental Disorders* (4th ed., text revision [*DSM–IV–TR*]; American Psychiatric Association [APA], 2000), is dependent on two components: *first*, in adults, a strong and persistent cross-gender identification; and in children, various behaviors like the insistence that they are the opposite sex, a preference for cross-dressing, and a preference for playmates of the opposite sex. The *second* component involves persistent discomfort with his or her sex or a sense of inappropriateness in the gender role of that sex. Various studies have tried to document the prevalence of the disorder, but there is no consensus on this issue (Bakker, van Kesteren, Gooren, & Bezemer, 1993; De Cuypere et al., 2007; Wilson, Sharp, & Carr, 1999). This study attempts to assess, among other things, to what extent laypeople understand the two previously named components of GID.

Inevitably, there has been considerable debate about the definition and the inclusion of this disorder in psychiatric manuals (i.e., the *DSM–V* [5th ed.; APA, 2013]; Cohen-Kettenis, 2001; Pressman, 1993; Winters, 2004). Compared to America, European academic and popular debate has essentially been on whether GID should be considered a disorder. British government papers (e.g., Lord Chancellor's Department Government Policy concerning Transsexual People, 2002) declared that GID was *not* a mental disorder, which has only stimulated popular debate and interest in the area.

An increased interest in the disorder (Campo, Nijman, Merckelbach, & Evers, 2003) has lead to various reviews being done (Zucker, 2005). Zucker and Bradley (1997) reviewed this research and noted themes from their interviews with children suffering from GID, which included a view of the world as threatening, the opposite sex being seen as offering protection and strength, and a fear of loss of caregivers. Boys with GID were significantly more disturbed than clinical controls, and possessed higher levels of both internalizing and externalizing psychopathology. When adolescents were examined, in addition to these findings, significant behavioral difficulties were found, as measured by maternal report.

More traits of separation anxiety were found in boys who met the complete criteria for GID, and this was consistent with the finding of increased levels of internalizing psychopathology from the Child Behavior Checklist (Zucker & Bradley, 1997). Increased levels of psychopathology have not only been found in affected individuals, but also in their parents. Higher rates of depression and borderline personality disorder were found in mothers of boys with GID, in comparison to the control group. Fathers have been far less well–studied, but it has been reported that higher levels of substance abuse were found in the fathers of 12 affected boys.

Many biological theories have been proposed with regard to the cause of GID (Zhou, Hofman, Gooren, & Swaab, 1995). Genetic research suggested there to is a *heritability component* to GID (Bailey, Pillard, R., Neale, M., & Agyei, 1993). Of course, the “nature versus nurture” distinction is a false dichotomy, as both are involved in behavioral development, although many laypeople (and some scientists) may speak or, indeed, think in “either/or” ways. One central question of this research is whether laypeople “lean toward” the one factor or the other in trying to explain GID.

This study is concerned with laypeople's beliefs about the causes and cures of GID. Its aim is partly to try to understand what laypeople think about GID and whether their beliefs about cause and treatment are related. Although one biological factor on its own does not appear to cause GID, there is evidence that biological factors have a partial capacity to explain the origin and development of GID.

In previous studies regarding lay beliefs about autism, parents supported biomedical models in relation to causes and treatments of autism (Furnham & Buck, 2003). Similarly, studies on lay theories of homosexuality showed the belief that it was often caused biologically or genetically (Furnham & Taylor, 1989). This study focused on beliefs about GID—clearly, a little known “disorder” among the general public, who may confuse it with other or different, if related, sexual disorders. It is important for those engaged in educating the public and reducing prejudice and discrimination toward those with GID to understand the nature and structure of lay beliefs about this condition so they can become better informed about where to target educational concerns (Hegarty & Golden, 2008).

It was hypothesized that, just as in many previous British lay theory studies, participants will favor psychosocial, rather than biomedical, explanations for causes and cures of GID (H1). Although studies into lay theories of GID have not been carried out before, studies into lay theories of homosexuality (Furnham & Taylor, 1989), autism, obsessive–compulsive disorder (Furnham & Buck, 2003), and sexual paraphilias (Twohig & Furnham, 1997) have all consistently shown that demographic variables, such as age and religiousness, predict lay beliefs about the different disorders. Because demographic factors are consistently related to lay theories of various problems, it was hypothesized that demographic variables (specifically, age and religious beliefs) will predict lay beliefs regarding GID (H2). In addition, it was hypothesized that, as in previous lay theory studies, positive correlations will be found between certain causes and cures factors of GID. Furnham and Rees (1988) hypothesized and demonstrated that clear relations were found between lay beliefs of causes and symptoms of schizophrenia (H3). This study involved a *qualitative* phase of interviews to construct a questionnaire that was used in the second main *quantitative* phase of the research.

## STUDY 1: THE PILOT STUDY

### Method

In the pilot study, 10 participants (5 men and 5 women) were given a semi-structured interview about their knowledge and understanding of GID. They were chosen to be as diverse as possible in demographic background (sex, age, and education). Eight were heterosexual and two were homosexual in terms of their sexual orientations. The age range was 22 to 61 years old, with an average age of 41.4 years (*SD* = 8.9). The interview was a 30- to 60-min, semi-structured interview mainly involving the cause, manifestation, and treatment of GID. Participants were initially provided with a brief, broad description of GID obtained from the *DSM–IV–TR* (APA, 2000), so they were adequately informed of the definition of the disorder. Participants were then asked to list as many possible factors that may *cause* GID. Second, they were asked for as many factors as possible that may *cure/treat* GID. Once it became clear that no new beliefs were being added, the interviews were terminated, and the start of the construction of the questionnaire begun. The idea of this small pilot study was to gather all commonplace, lay ideas about GID expressed in non-technical language. Inevitably, a bigger quantitative study with a more heterogenous population may have offered a few more ideas.

### Results

During the 10 interviews, a variety of causes were suggested by participants, with the most popular being hormonal imbalances, suggested by seven of the participants. Environmental factors, such as society stereotypes, and cultural expectations were mentioned by one-half of the participants, and both genetic and parental factors were mentioned by four participants. Overall, one-half of the participants mentioned congenital damage as a possible cause, with two individuals referring to brain damage as a cause, two to brain dysfunction, and one to his or her mother's illness during pregnancy. The majority of participants mentioned both biological (hormonal, genetic, and congenital factors) and environmental or parental factors when interviewed regarding the causes of GID.

There was less variability in possible cures/treatments mentioned by participants. Medical therapies and behavioral therapies were suggested by eight participants, respectively. Only one participant suggested there was “no actual cure” for GID. Also, hypnosis, removal or change of the affected individual's environment, and adapting social roles of the opposite sex to biological ones were each suggested by one participant. In general, participants were uncertain and hesitant with their responses, with many stating, “I really don't know much about GID,” although a definition was provided. This is not unexpected given the rarity of the disorder.

The interview responses were then used to create the questionnaire. However, previous research suggested some other beliefs that were thought to be relevant; thus, the statements obtained from the interviews were also supplemented with statements participants suggested in the study (Furnham & Buck, 2003): “lay beliefs about autism and obsessive–compulsive disorder” (Furnham & Buck, 2003) and “lay beliefs about overcoming fetishism, paedophilia, sexual sadism, and voyeurism” (Twohig & Furnham, 1997). Once the attitude statements were collected and written, they were piloted for their clarity and extreme responding (which may lead to floor and ceiling effects). Various changes were made at this stage.

## STUDY 2: THE MAIN STUDY

### Method and Participants

In all, 124 questionnaires were completed out of a total of 134 distributed in central London (92%). There were 57 men, 66 women, and 1 was unknown. The age range of participants was between 20 and 62 years, with a mean age of 39.25 years (*SD* = 14.68). The majority of participants were married (60%). There was some variation in ethnicity, with 75% of participants being Caucasian, 16.1% Asian (subcontinent), 4% Asian (Far East), and 4.8% classifying themselves as “other.” All participants were educated to at least the General Certificate of Secondary Education level (10th grade in the United States; 2.4%), with 62.7% being educated at a degree level or higher. Some participants (22.6%) described themselves as being very religious, 6.5% as only being religious with parents or family, 22.6% believed in religion but did not practice, 16.1% were hardly observant, and 30.6% stated they were not at all religious. Strongly leftwing views were held by 4.8%, somewhat leftwing by 11.3%, more leftwing than rightwing by 20.2%, neither left- or rightwing by 32.3%, more rightwing than leftwing by 23.4%, and somewhat more rightwing than leftwing by 7.3%. The occupations held by participants were divided into four groups: student (23%), teacher (14%), engineer (23%), and “other” (29%). The sexual orientation of the sample was not assessed, which is a possible limitation. This was clearly a non-representative, convenience sample, which makes generalization to the wider population problematic.

### Questionnaire

The questionnaire first provided a brief description of GID so that participants had a clinical description and definition of the disorder:

Gender identity disorder is a conflict between a person's actual physical gender and the gender that person identifies himself or herself as. For example, a person identified as a boy may actually feel and act like a girl. The person experiences significant discomfort with the biological sex they were born.

Participants were then asked to complete demographic details regarding their age, sex, civil status, ethnicity, religious view, educational level, occupation, and political view. Declarative statements were then presented under the headings of *causes* and *cures/treatments* (e.g., “GID is caused by the affected individual's negative self-image”; see Tables 1 & 2). Participants were asked to declare to what extent they agreed with each statement on a scale ranging from 1 (*strongly agree*) to 7 (*strongly disagree*).

**TABLE 1:** Means for Questionnaire Items for Causes of Gender Identity Disorder and Factor Loadings

Item	*M*	*SD*	Rank (mean)	1	2	3	4
12. Bullying at school	4.53	1.71	14	.784			
10. Physical/mental abuse as a child/adolescent	3.58	1.75	4	.767			
18. Experiencing a disturbed family upbringing	3.75	1.68	6	.765			
21. Affected individual's negative self-image	3.82	1.64	7	.761			
20. Breakdown of relationship with same-sex parent	4.72	1.61	16	.760			
7. Traumatic experiences in life	3.36	1.74	3	.753			
19. Peer group rejection by
children of same biological sex	4.18	1.66	10	.723			
11. Dislike of society gender stereotypes	4.39	1.65	11	.530			
3. Mother experiencing an illness during pregnancy	4.79	1.51	17		.774		
13. Complications during pregnancy	5.58	3.84	20		.763		
5. Mother abusing alcohol/drugs during pregnancy	4.48	1.60	13		.737		
6. Brain damage of affected individual	4.55	1.71	15		.727		
8. Brain dysfunctioning of affected individual	3.88	1.71	8		.534		
2. Religious/cultural beliefs in environment (e.g., belief that females are the “weaker” sex)	4.92	1.76	18			.807	
15. Greater believed economic benefits of opposite sex	5.42	5.83	19			.692	
1. Family members wished for a child of the opposite sex	4.40	1.74	12			.610	
14. Environmental upbringing with members of the opposite sex	3.95	1.68	9			.497	
16. Genetic abnormalities (e.g., chromosomal)	2.48	1.58	1				.776
4. Hormonal imbalances in affected individual	2.64	1.53	2				.680
17. Affected individual is homosexual	3.73	1.86	5				.421
9. Food allergies in affected individual	6.35	3.73	21				.800

*Note.* Scale ranges from 1 (*strongly agree*) to 7 (*strongly disagree*).

**TABLE 2:** Means for Questionnaire Items for Cures of Gender Identity Disorder (GID) and Factor Loadings

Item	*M*	*SD*	Rank	1	2	3	45
5. Psychological therapy to accept their childhood	4.11	1.77	7	.820			
7. Therapy to explore their issues	3.91	1.65	6	.818			
13. Counselling	3.51	1.76	3	.762			
9. Adapting social roles of opposite sex to biological one	4.35	1.59	8	.627			
11. Removal/change of environment	4.63	1.67	11	.610			
8. Providing a warm and loving environment	3.59	1.82	4	.569			
16. Affected individuals willpower to be “normal”	4.42	1.73	9	.543			
12. Change of diet in affected individual	5.72	1.46	17		.711		
10. Encouraging interaction with only other “normal” individuals	5.00	1.71	12		.698		
17. Lobotomy (brain surgery)	5.59	1.69	16		.624		
18. Affected individual's institutionalization	5.89	1.53	18		.596		
14. Rewarding normal behavior can cure GID	5.26	1.61	14		.565		
2. Hypnosis	4.55	1.53	10			.729	
4. Affected individual's luck	5.02	1.59	13			.639	
15. No actual cure	3.07	1.65	1				−.700
6. A belief in God can help someone overcome GID	5.46	1.89	15				.566
1. Hormone replacement	3.36	1.65	2				.798
3. Gender reassignment surgery	3.71	1.64	5				.566

*Note.* Scale ranges from 1 (*strongly agree*) to 7 (*strongly disagree*).

### Procedure

The questionnaire was anonymously completed by participants in their own time. They were requested to read the introductory statements consisting of the definition of GID. They were then requested to return the questionnaire to the researcher, or a researcher's assistant, and thanked for their participation. Anonymity was assured to all participants who were interviewed and completed the questionnaire. They were essentially a convenience sample, rather than a representative sample, of the population. Ethical permission was sought and granted from the appropriate university department committee where we worked.

### Results

Table 1 also shows the mean ratings for each question on the causes sections of the questionnaire and the ranked position of each mean (1 = *strongly agree*, 4 = *no opinion*, 7 = *strongly disagree*). There was a large range of means (2.48–6.35) and considerable variability of means, which suggests that participants believed in certain causes more strongly than others, and viewed such as more accurate. For instance, “genetic abnormalities (e.g., chromosomal)” was the most popular cause (2.48), followed by “hormonal imbalances in individual” (2.64). “Food allergies in affected individual” was the least popular cause (6.35), and was the only item that did not load onto any of the four factors.

Factor analysis with orthogonal rotation (varimax) was used to examine the underlying dimension of the lay beliefs examined in this study. The eigenvalue >1.00 rule was used, although other criteria could have been chosen (e.g., scree test). Four factors were extracted from the causal beliefs and are shown in Table 1, which accounted for 61.49% of the variance. The four factors consisted of items with loadings >0.4. In cases where an item loaded onto more than one factor, it was included within the factor for which it had the largest loading.

The first factor (Items 12, 10, 18, 21, 20, 7, 19, and 11) was labelled *upbringing and personal factors*, as they mainly related to issues surrounding the individual's upbringing (e.g., “experiencing a disturbed family upbringing”) and certain factors related to the individual. The second factor (Items 3, 13, 5, 6, and 8) was labelled *pregnancy and brain abnormalities*—the factors related to the mother's pregnancy and disorders of the affected individual's brain. The third factor (Items 2, 15, 1, and 14) was given the label *beliefs about gender*, and included items that were present in the affected individual's environment and which were often out of their control. The fourth and final factor (Items 16, 4, and 17) was called *genetic and physiological*, as it mainly related to issues regarding the individual's biological makeup.

There was also a considerable variation in participants’ agreement to the cures listed in the questionnaire, although this was less marked than for causes. The range of means was 3.07 to 5.89, with participants showing the greatest agreement to “no actual cure” (3.07) and the least agreement to “affected individual's institutionalization” as a cure (5.89). The results for cures of GID were analyzed separately; and, once again, factor analysis with orthogonal rotation (varimax) was used. Five factors emerged, which accounted for 65.11% of the variance (see Table 2). Items that had loadings of >0.4 were selected for inclusion in each of the five factors. The first factor (Items 5, 7, 13, 9, 11, 8, and 16) was labelled *psychological assistance and personal factors*, as it related to therapy and an individual changing aspects of his or her life. The second factor was labelled *extreme medical and behavioral changes*, as it included items such as “institutionalization” and changing whom the affected individual interacted with. *Alternative therapies* was the label added to the third factor, as it included hypnosis and an individual's luck as its items. The fourth factor was labelled *external factors*, and consisted of the following items: “no actual cure” and a “belief in God can help someone overcome GID.” *Medical treatments* was the label applied to the fifth factor, and consisted of “hormone replacement therapy” and “gender reassignment surgery,” which are treatments that are in practice today.

To see if there was a “logical” relation between perceived cause and cure, correlations were computed between all factor scores. Table 3 shows correlations between different causal and cure factors obtained from the factor analysis. The highest correlation, *r* = .61, was between the causal factor *upbringing and personal factors* and the cure factor *psychological assistance and personal factors.* Other cause and cure factors that correlated at the *p* < .01 level were *pregnancy and brain abnormalities* with *extreme medical and behavioral changes* (*r* = .45) and *biomedical treatments* with *medical treatments* (*r* = .39).

**TABLE 3:** Correlation Between Cause and Cure/Treatment Factors

	Cure/treatment factors				
Cause factors	1. Psychological assistance and personal factors	2. Extreme medical and behavioral changes	3. Alternative therapies	4. External factors	5. Medical treatments
1. Upbringing and personal factors	.61**	.03	.20	.20	−.06
2. Pregnancy and brain abnormalities	−.02	.45**	.22*	.18	.05
3. Beliefs about gender	.16	.25*	.22*	.01	−.03
4. Genetic and physiological	.21*	−.11	−.19	−.08	.39**

Note. *p<.05; **p<.01.

At the *p* < .05 level, there were four instances where a cause factor correlated with a cure factor: *pregnancy and brain abnormalities* with *alternative therapies* (.216), *environment* with *extreme medical and behavioral changes* (.253) and with *alternative therapies* (.215), and *biomedical* with *psychological assistance and personal factors* (.213).

A regression analysis was performed to see whether the participants’ demographic details (age, sex, religious status, educational level, and political views) reflected their beliefs. Nine regressions were computed (1 for each factor score), but none were significant.

## DISCUSSION

The results do not support H1, and show that participants generally favored biomedical factors as perceived causes and cures/treatments of GID. This may, in part, reflect the way in which neuroscience has penetrated popular culture and lay understandings of behavioral issues. In the causes section, “genetic abnormalities” and “hormonal imbalances” were ranked 1 and 2, respectively. In the cures section, there was slightly more variation, as “no actual cure” was ranked as top, “hormonal replacement” was featured second, and “gender reassignment surgery” was ranked fifth. Reassuringly, methods that are no longer in practice due to their unethical nature, such as “lobotomy” (Item 17) and “affected individual's institutionalization” (Item 18), were ranked at 16 and 18, respectively. Participants agreed that upbringing may have an impact on causing GID, and factors related to this were ranked at 4 and 6. In relation to cures of GID, “counselling” was popular among participants, and was ranked third, and “psychological therapy to accept their childhood” was ranked sixth.

A preference for biomedical causes and cures may be due to the idea that there is no moral outcome from the preference of a biomedical model, although there may be considerable implications associated with this preference. Although no prior studies of lay beliefs regarding GID appear to have been carried out prior to this study, a few such studies examined homosexuality. Homosexuality was removed from the *DSM–II* (2nd ed.; APA, 1968) in 1973, but many still see homosexuality and GID as being interrelated. This link was also suggested by the questionnaire where, in the cause section, the item “affected individual is homosexual” was ranked 5th out of 21 items.

Furnham and Taylor (1989) conducted a study into lay beliefs of homosexuality and found that participants thought the most important aetiological factors were *problems associated with early relationships, genetic differences*, and *fear of women.* With regards to cures, participants mostly believed in the efficacy of hormonal treatment, followed by psychotherapy, but far less in surgery. Therefore, the popular choices of *genetic differences* as a cause and *hormonal treatment* as a cure are similar to the findings of this study; therefore, it can be inferred that there is some link between lay beliefs of homosexuality and GID. The results on lay theories of homosexuality have recently been replicated (Furnham & Saito, 2009).

The term “psychological essentialism” refers to ascribing a fixed, underlying nature—or essence—to category members (Medin & Ortony, 1989). Essentialists believe that biology grounds sexual orientations, and that these orientations are unlikely to change and are unaffected by cultural shaping (Haslam & Levy, 2006). Although this study covers the area of GID, the preference of participants for a biological basis of GID suggests a tendency toward essentialist beliefs. Essentialism can be used to define all category members, although its nature may be obscure to the individual who ascribes it and can result in category members being seen as “fundamentally alike” and having inferences drawn regarding them.

Many researchers pointed out negatives associated with essentialist beliefs. Early researchers found essentialism as being a fundamental component of prejudice, and others have suggested that these beliefs can legitimize and naturalize unequal social arrangements (Yzerbyt, Rocher, & Schadron, 1997). In addition, Martin and Parker (1995) found that a greater endorsement of racial and gender stereotypes occurs if there is an essentialist belief regarding the biological basis of gender and race. The research of Haslam and Ernst (2002) regarding beliefs about sexual orientation and prejudice suggested that essentialism does not have negative connotations and that a belief in the immutability, biological basis, historical, and cross-cultural universalism leads to greater tolerance. However, the presence of beliefs in immutability and discreteness is fundamentally associated with prejudice (Haslam & Levy, 2006). Herek and Capitanio (1995) also found an association between a belief in the biological basis of homosexuality and its uncontrollability, as it predicted a positive attitude toward gay and lesbian people. Whether these theories of essentialist beliefs can be fully generalized to GID is unknown, but in the presence of inadequate research, the findings of research into homosexuality beliefs can provide possible explanations and models for research.

H2—demographic variables predicting lay beliefs regarding causes and cures of GID—was not confirmed, as demographic variables did not account for any of the variability of the results. It was thought likely that sexual disorders have moral overtones, which mean they are related to religious and political values. However, surprisingly and inexplicably, religion was not related to beliefs about cause or cure.

H3—there would be a correlation between certain cause and cure/treatment factors—was partially upheld. The highest correlation was unsurprisingly found between the cause factor *upbringing and personal factors* and the cure/treatment factor *psychological assistance and personal factors.* Also, a significant correlation was found between *pregnancy and brain abnormalities* and *extreme medical and behavioral changes*, along with one between *genetic and physiological causes* and *medical treatments.* This suggests that those participants who favored environmental factors as causes of GID also favored environmental factors as a cure, and likewise for those who favored *genetic and physiological causes.* However, a positive correlation was found between *biomedical causes* and *psychological assistance and personal factors* as cure/treatment factors, and *environment causes* and *extreme medical and behavioral changes* as cures. In this sense, people may favor complex, multi-causal theories of the origin of GID. These findings, in relation to correlates between causes and cures, were similar to those of Furnham and Taylor (1989) regarding lay beliefs of homosexuality.

The ranking of “gender reassignment surgery” as 5th out of the 18 items in the cure/treatment section suggests that study participants considered it, generally, a potential cure/treatment. In practice, however, the majority of individuals who are diagnosed with GID do not have surgery (Zucker & Bradley, 1997).

There is a great scope for future research into lay theories of GID, and this must be considered a pilot study with self-evident limitations. Future studies could aim to use a wider range and larger number of participants. This was certainly far from a representative sample, which inevitably threatens the generalizability of these results and require replication. It would be interesting to look at those with GID themselves, their relatives, as well as those in sex therapy research. Many subgroups could be studied to determine their knowledge and the precise determinants of accepting, positive, and tolerant versus rejecting, negative, and intolerant attitudes. The questionnaire could be adapted to contain all the various factors proposed to cause and cure GID from the research of various psychologists and psychiatrists. Further, it is important to explore whether participants’ ideas about cause and cure are related to such issues as the age of GID people (children vs. adolescents), gender (males vs. females), or early versus late onset GID. Also, studies into essentialist beliefs regarding GID could be carried out in a similar form to the study by Haslam and Levy (2006). Also, cross-cultural comparisons could be made, as Newman (2002) found that culture influenced the presentation and understanding of gender dysphoria and gender-aberrant behavior.
